# Molecular Characterization of Uropathogenic *Escherichia coli* Reveals Emergence of Drug Resistant O15, O22 and O25 Serogroups

**DOI:** 10.3390/medicina55110733

**Published:** 2019-11-11

**Authors:** Ruta Prakapaite, Frederic Saab, Rita Planciuniene, Vidmantas Petraitis, Thomas J. Walsh, Ruta Petraitiene, Rasa Semoskaite, Rasa Baneviciene, Lilija Kalediene, Povilas Kavaliauskas

**Affiliations:** 1 Life Sciences Centre, Vilnius University, Vilnius 10257, Lithuania; prakapaite.ruta@gmail.com (R.P.); lilija.kalediene@gf.vu.lt (L.K.); 2 Institute for Infectious Diseases and Pathogenic Microbiology, Prienai 59115, Lithuania; freddysaab@hotmail.com (F.S.); vip2007@med.cornell.edu (V.P.); rop2016@med.cornell.edu (R.P.); 3 Institute for Microbiology and Virology, Lithuanian University of Health Sciences, Kaunas 47181, Lithuania; rplanciuniene@gmail.com; 4 Biological Research Center, Lithuanian University of Health Sciences, Kaunas 47181, Lithuania; 5 Division of Infectious Diseases, Department of Medicine, Weill Cornell Medicine, New York, NY 10065, USA; thw2003@med.cornell.edu; 6 National Laboratory for Public Health Surveillance, Kaunas 44153, Lithuania; Rassemosk@gmail.com (R.S.); rasyte.g@gmail.com (R.B.)

**Keywords:** *E. coli*, Lithuania, serogroups, UPEC, urinary tract infections

## Abstract

**Background and Objectives:** Uropathogenic *Escherichia coli* (UPEC) are common pathogens causing urinary tract infections (UTIs). We aimed to investigate the relationship among clinical manifestation, serogroups, phylogenetic groups, and antimicrobial resistance among UPEC. **Materials and Methods**: One-hundred *Escherichia coli *isolates recovered from urine and ureteral scrapings were used for the study. The prevalence of antimicrobial resistance was determined by using European Committee on Antimicrobial Susceptibility Testing (EUCAST) recommendations. *E. coli *serogroups associated with UTI, as well as phylogenetic diversity were analyzed using multiplex PCR reactions. **Results:** Eighty-seven strains (87%) were isolated from females, while 13 (13%) from males. A high frequency of resistance to cephalosporins (43%) and fluoroquinolones (31%) was observed. Among UTI-associated serogroups O15 (32.8%), O22 (23.4%), and O25 (15.6%) were dominant and demonstrated elevated resistance rates. The *E. coli *phylogenetic group B2 was most common. These observations extended to pregnant patients with asymptomatic bacteriuria. **Conclusions:** Due to high rates of resistance, strategies using empirical therapy of second-generation cephalosporins and fluoroquinolones should be reconsidered in this population.

## 1. Introduction

Extraintestinal *Escherichia coli* (ExPEC) is considered to be the most common Gram-negative infectious agent causing extraintestinal infections [1]. The ability of ExPEC to cause infections is determined by virulence factors and host susceptibility to infection [2]. ExPEC can express combined pathogenicity pathotypes and utilize different virulence strategies in order to cause various clinical manifestations.

As a common pathotype of ExPEC, uropathogenic *E. coli* (UPEC) mainly originates from intestinal microbiota and can be attributed to the major cause of 75–95% cases of uncomplicated urinary tract infections (UTI), that affect approximately 150 million people worldwide annually [3,4]. High frequency and recurrence of UTI demand extended usage of antibiotics that leads to the selection of resistance to the last-line antibiotics [5], as well as the rise of virulent UPEC clones [6,7,8]. An untreated or mistreated UTI can ascend to kidneys and cause systemic infections with high mortality rates.

Various *E. coli* serogroups have been previously found to be more common between *E. coli* isolated from cases of UTI [9,10]. However, little is known about the relationship among antimicrobial resistance and serogroup, as well as their relationship to phylogenetic groups and infection types. *E. coli* is phylogenetically classified into seven main groups: A, B1, B2, C, D, E, and F [11]. Pathogenic ExPEC strains typically belong to B2 and D groups [12,13], while groups A and B1 are usually associated with commensal strains [14]. B2 and D groups are also considered to be more virulent and susceptible to antibiotics in comparison to those of A and B1 groups [15,16]. 

Within Baltic region countries little is known about the spectrum of infections caused by UPEC, their molecular epidemiology or patterns of resistance. We therefore aimed to investigate the relationship among clinical manifestation, serogroups, phylogenetic groups, and antimicrobial resistance among UPEC in Lithuania.

## 2. Materials and Methods

### 2.1. Escherichia coli Isolates

The study was approved by Institutional Review Boards of the Lithuanian Health Sciences University (No.: BMC-LMB(M)-310,2019-02-08). A collection of total 100 clinically unrelated *E. coli* strains, isolated from urine (*n* = 98) samples and ureteral (*n* = 2) scrapings were used for this study. Isolates were collected during the period of 2017–2018 from seven medical centers in five different cities of Lithuania. Isolates were recovered from previously anonymized patients undergoing routine medical examination. All received strains were confirmed as *E. coli* by biochemical tests (RapID ONE System, ThermoFisher Scientific) and were stored at −80 °C in commercial cryopreservation media (Prolab Diagnostics).

### 2.2. Study Groups

Using the anonymized clinical data provided, all isolates were classified into three groups that were used for further analysis: infectious group (isolates from patients with clinically confirmed UTI and endometritis), non-infectious group (isolates from female patients with asymptomatic bacteriuria; ASB), and unknown diagnosis group (isolates from female and male patients with unavailable medical records). ASB was considered when patients had no clinical symptoms, but two consecutive urine cultures were positive (≥10^5^ CFU/mL) for *E. coli*. The term urinary tract condition (UTC) was used in this study to describe any involvement of a UPEC in the urinary tract. 

### 2.3. Evaluation of Antimicrobial Susceptibility

Antimicrobial susceptibility to 11 antibiotics was evaluated by disk diffusion method and interpreted by European Committee on Antimicrobial Susceptibility Testing (EUCAST) standard [17]. Susceptibility to ampicillin (AMP), amoxicillin/clavulanate (AMC), cefuroxime (CXM), ciprofloxacin (CIP), amikacin (AK), gentamicin (CN), tobramycin (TOB), nitrofurantoin (F), trimethoprim (TMP), imipenem (IPM), and meropenem (MEM) was evaluated. Antibiotic discs were purchased from Liofilchem.

### 2.4. Preparation of Template DNA

Template DNA was prepared by thermal lysis method [18] and used for all PCR reactions. Briefly, 2–3 colonies of *E. coli* were suspended in 300 μL of nuclease-free water. Samples were incubated at 100 °C water bath for 10 min and centrifuged at 10,000 × *g* for 10 min at 4 °C. Prepared lysates were stored at −20 °C.

### 2.5. Serogrouping of E. coli Isolates by Using Multiplex PCR

*E. coli* serogroups associated with uropathogenicity (O1, O2, O4, O6, O7, O8, O15, O16, O18, O21, O22, O25, O75, O83) were determined by multiplex PCR, as described by Li et al. [19]. Two separate PCR mixes, containing approximately 50–100 ng of template DNA (3 μL), were used for molecular serogrouping. They were prepared, as described by Li et al., by mixing 1× Taq buffer with KCl (ThermoFisher Scientific), 2.5 mM MgCl_2_, 0.3 μM of each deoxynucleotide (dNTP) (ThermoFisher Scientific), 0.05–0.13 μM of the respective primers (Metabion), 2 U of Taq DNA polymerase (ThermoFisher Scientific), and adding nuclease-free water until a final volume of 30 μL.

### 2.6. Phylogenetic Grouping of E. coli Isolates

Phylogenetic grouping was performed by using multiplex PCR, as described by Clermont et al. [20]. Reaction was performed in 20 μL of PCR mix containing 200 ng of template DNA. PCR mix was prepared, as described above, by mixing 1× Taq buffer with KCl (ThermoFisher Scientific), 1.25 mM of MgCl_2_, 2 μM of each dNTP (ThermoFisher Scientific), 1 μM of primers (Metabion), 2.5 U of Taq polymerase (ThermoFisher Scientific), and nuclease-free water.

### 2.7. Statistical Analysis

De-identification of patient data was performed using Caristix HL7 software at each of the primary collaborating centers before submitting the isolates to the laboratory. R statistical software (version 3.5.3) was used for analyzing and modeling the data. Binomial testing was used for comparing the proportions of females versus males among the samples pertaining to *E. coli* urinary tract hosts. The chi-square test was used for analysis of differences in proportions for categorical variables. Ages were expressed in boxplots as means, interquartile ranges, and ranges. Data was considered significant when *p* < 0.05.

## 3. Results

### 3.1. Prevalence of E. coli-Caused Conditions 

**Figure 1:**
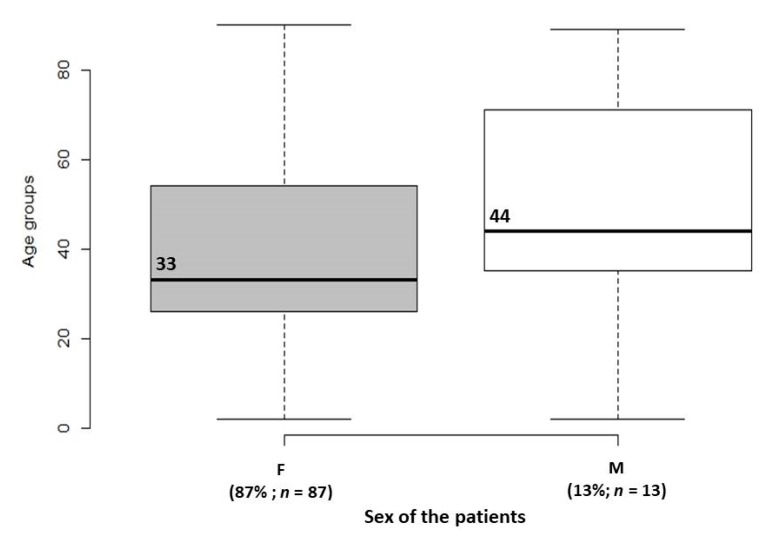
The age distributions 87 female (F) and 13 male (M) patients with *E. coli* caused conditions. The median age of female patients was 33 years while 44 years was the median age among male patients with *E. coli* caused conditions. The whiskers in both boxplots indicate lowest and highest age among females (2–90 years) and males (2–89 years).

In total, 98 strains were isolated from urine samples and two strains were isolated from uterine scrapings. Eighty-seven *E. coli* strains (87%) were isolated from females; whereas, 13 strains (13%) were isolated from males (Figure 1). *E. coli*-caused conditions were more common among females, than males (*p* < 0.05) in all study groups and demonstrated the sex specific predisposition to infection.

Among 100 isolates, 53 isolates (53%) were recovered from infectious study group patients. Thirty-two were recovered from non-infectious study group patients, and 15 were obtained from patients with unavailable clinical records. The most commonly observed infectious study group (53 cases) consisted of 20 confirmed UTI (37.7%), 19 with pyelonephritis (35.8%), 10 with cystitis (18.9%), two of endometritis and two of pyelonephritis with complications of urosepsis (3.8%), respectively (Table 1). 

Thirty-two *E. coli* strains (32%) were isolated from pregnant women, who participated in a prenatal surveillance program. All strains caused ASB at the time of culture. One patient later developed an unspecified UTI.

### 3.2. Age Distribution among Patients with E. coli-Caused UTC

Among the 87 females, the age of the patients ranged from 2 to 90 years. Among 13 males, the age ranged from 2 to 89 years. The median age of females was 33 years, while observed median age among males was 44 years (Figure 1).

### 3.3. Antimicrobial Susceptibility Patterns among UPEC

Among all *E. coli* strains, 86% showed resistance to at least one tested antimicrobial, while 22% of strains were resistant to three or more antibiotics. A high frequency of resistance to cephalosporins was observed; e.g., as 43% of strains was resistant to cefuroxime (Table 1). Lower levels of resistance to penicillins were observed in comparison to that of cephalosporins; 32% of all tested isolates were resistant to ampicillin and 28% of *E. coli* strains showed resistance to amoxicillin/clavulanate. Ciprofloxacin resistance was detected in 31% of all isolates. Lastly, 16% of isolates showed resistance to trimethoprim. Lower resistance rates to aminoglycosides were observed during this study. The highest rates of resistance among tested aminoglycosides were observed to tobramycin (6%), and followed by amikacin (4%) and gentamicin (3%). Among tested isolates, 3% showed resistance to nitrofurantoin. Furthermore, all isolates were susceptible to carbapenems (meropenem and imipenem) (Table 1). Additionally, intermediate resistance to antibiotics was observed. In general, 25% of all tested *E. coli* demonstrated intermediate susceptibility to at least one antimicrobial. Nine percent showed intermediate susceptibility to tobramycin, 6% to amikacin, and 5% to trimethoprim (Supplementary Table S1).

### 3.4. Serological Diversity among E. coli Strains Isolated from Patients with UTC 

Among 100 isolates, 64% of *E. coli* isolates belonged to a serogroup associated with uropathogenicity (O1, O2, O4, O6, O7, O8, O15, O16, O18, O21, O22, O25, O75, O83), while the serogroup of the remaining 36% tested *E. coli* isolates was not identified, hence belonged to other serogroups (Table 2). Among UTI-associated serogroups, O15 (32.8%) was the most prevalent and followed by O22 (23.4%), and O25 (15.6%) respectively. 

Among female isolates representing UTI-associated serogroups, O15 (95.2%), O22 (86.7%), and O25 (100%) were the most commonly detected. Among isolates recovered from male patient population (*n =* 13), two isolates, were identified as *E. coli* O22 thus classified as a UTI-associated serogroup (Table 2). 

**Figure 2:**
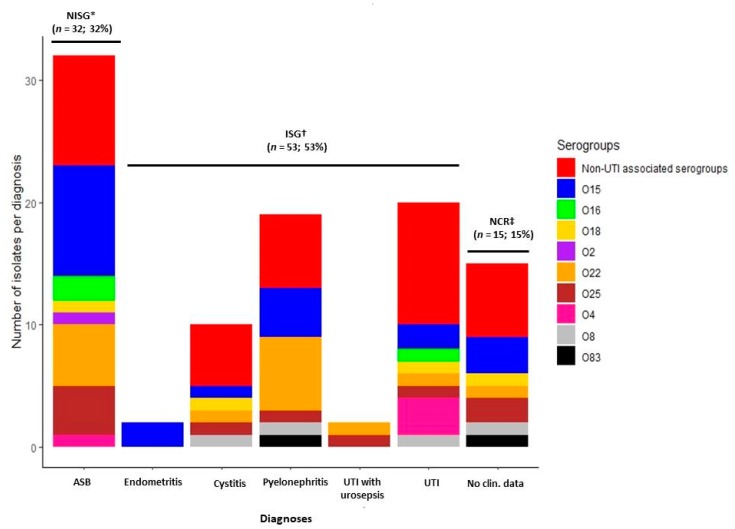
The distribution of *E. coli* UTI-associated and non-UTI associated serogroups among different clinical conditions. NISG* = non-infectious study group; ISG† = infectious study group; NCR‡ = group with no clinical records available. Abbreviations of legend: ASB = asymptomatic bacteriuria; UTI = clinically confirmed urinary tract infection.

Serogroups O15 and O22 (17%) were the most prevalent among 53 *E. coli* isolates obtained from the infectious study group (Figure 2). Serogroups O8, O15, O18, O22, and O25 of isolates from the infectious group with clinically proven cystitis, represented a similar distribution of UTI-associated serogroups (1.9%), while the majority of the isolates represented non-UTI associated serogroups (9.4%). Among patients with pyelonephritis, serogroups O15 and O22 were the most prevalent and represented 7.6% and 11.3% of all isolates, respectively (Figure 2).

In the cases within the non-infectious study group, serogroups O15 (28.1%) and O22 (15.6%) were the most common isolates obtained from patients with ASB. However, serogroups of 28.1% of the isolates were not identifiable by the assay used (Figure 2).

### 3.5. Increased Multidrug Antimicrobial Resistance Observed among O15 Serogroup 

Increased antimicrobial resistance levels were observed among *E. coli* UTI-associated serogroups in comparison to those of non-UTI associated serogroups. Increased resistance frequencies were observed among serogroups O15, O22, and O25. Those serogroups had demonstrated extensive resistance rates (resistance to two and more antibiotics) regardless of the diagnosis. Of O15, 57.1% of isolates indicated resistance to ciprofloxacin, and 71.4% were resistant to ampicillin. Of *E. coli* O22, 60% was resistant to ciprofloxacin (Table 2). When compared to other UTI-associated serogroups, O15 showed significantly higher resistance (*p =* 0.016) to ampicillin and trimethoprim (*p =* 0.027) and was resistant to more than one antimicrobial agent.

### 3.6. Phylogenetic Diversity among E. coli Isolates

Phylogenetic group B2 was the most prevalent (50%) among the analyzed population of *E. coli*, followed by group D that represented 25% of all isolates. Furthermore, 18% of isolates represented B1, while 7% was grouped as A. A similar proportion of distribution among *E. coli* phylogenetic groups was observed when isolates obtained from female patients were analyzed. Phylogenetic group B2 isolates were also highly prevalent among the smaller group of males (Table 3). 

*E. coli* B2 was dominant (54.7%) and followed by D (24.5%) in the infectious group. Group B2 was prevalent between both sexes in the infectious group (Table 3). The different distribution was observed in the non-infectious group, as group A was considerably more prevalent (12.5%) in comparison to those of other groups and conditions. Groups D and B2 were the most common and were followed by low frequencies of group A in the group of unavailable medical data.

**Figure 3:**
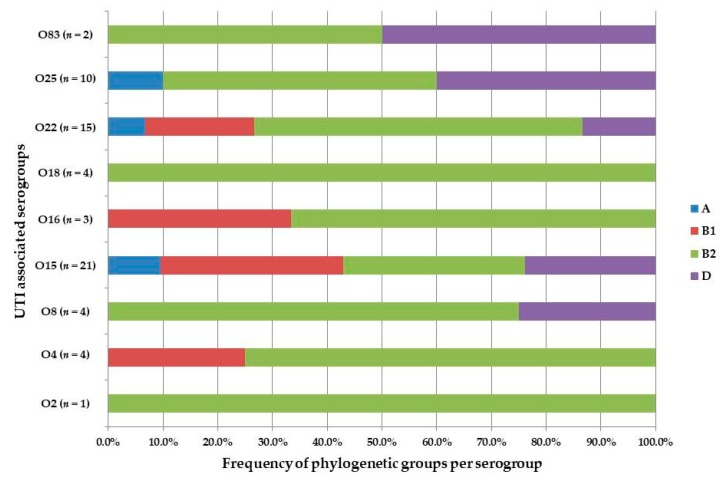
The distribution of *E. coli* phylogenetic groups (A, B1, B2, and D) among UTI associated serogroups. *E. coli* serogroups O1, O6, O7, O21, and O75 was not detected so therefore not included in the figure.

Phylogenetic group B2 was common among serogroup O22 (60%) and O25 (50%) isolates (Figure 3).

### 3.7. Patterns of Antimicrobial Susceptibility among E. coli Phylogenetic Groups

Group B1 isolates showed highest resistance to ciprofloxacin (44.4%), while group A reached 28.6%, and was followed by B2 and D (28.0% and 28.0%, respectively). Highest resistance rates to amoxicillin/clavulanate were observed among group D (48.0%), while B1 and B2 reached 22.2% and 22%, respectively. Group A demonstrated lowest resistance rates (14.3%). Increased resistance to ampicillin was observed among the members of group A (42.9%) and D (36.0%), whereas B1 and B2 demonstrated 27.8% and 30.0% resistance. Isolates of A, D, B2, and B1 groups were highly resistant to cefuroxime (57.1%, 52%, 40%, and 33.3%, respectively) (Table 4). There were no significant differences between phylogenetic groups and association between bacteriuric and non-bacteriuric patient populations. 

## 4. Discussion

The study reflects current prevalence and variety of urinary tract associated conditions, caused by *E. coli* in Lithuania, a Baltic region country with the estimated size of 2.8 M population. This study highlights the phylogenetic composition of *E. coli* as well as the rates of antimicrobial resistance and associations of resistance, phylogenetic group, and serogroup with clinical condition. An increased resistance to *β*-lactams and fluoroquinolones was demonstrated in this study. The overall levels of resistance to those particular antibiotics call for better consideration for usage of cephalosporins and fluoroquinolones for empiric therapy in Lithuania. We also demonstrated that *E. coli* caused condition-specific serogroup composition in Lithuania. For the first time, the high distribution of multidrug resistant *E. coli* serogroup O15 was demonstrated among Lithuanian patients.

This studied population in Lithuania demonstrated a wide age distribution from 2 to 90 years. The predominant age group among females with a clinically confirmed infectious process (UTI and endometritis) was 20–39 years. The etiology of UTI among young women might be influenced by intercourse related issues, such as frequent sexual activity, new sex partner, and the usage of spermicides [21,22]. However, an increased incidence of UTI was also observed among older females, aged from 60 to 79 supporting the hypothesis that age is an important factor for predisposition to UTI [23]. 

A lower frequency of UTI and associated conditions among male patients was observed. UTI and associated conditions were more common among older males, ranging from 40 to 59 years, in comparison with females (Figure 1). 

Asymptomatic bacteriuria (ASB) is a common condition in various populations [24,25,26]. Pregnancy, genitourinary abnormalities or indwelling urinary devices increase the risk of developing ASB that could potentially lead to severe complications. In this study, ASB was not observed among other patients, except pregnant women. Increased ASB prevalence during pregnancy can be explained by an increased susceptibility to infections [27]. Thus, during pregnancy ASB can possibly develop to complicated UTI with life threatening complications. ASB in pregnancy is an independent risk factor for preterm delivery [28]. Moreover, ASB might possess a serious threat to pregnant women and even to neonates, as vertical transmission of UPEC might provoke early-onset neonatal infections [29]. However, treatment of ASB is also associated with a higher frequency of antibiotic resistance [30]. 

Our data indicate that phylogenetic groups, serogroups, and patterns of antimicrobial resistance are similar in the ASB population and infectious group. These patterns include a relatively high level of antimicrobial resistance, particularly among isolates of serogroup O15. Thus, emerging antimicrobial resistance poses challenges even among pregnant patients with ASB. Therefore, great attention should be focused on monitoring prevalence, serological diversity, and susceptibility patterns of *E. coli-*caused ASB during the pregnancy period.

Rising antimicrobial resistance is a global problem. In our study, a high distribution of antimicrobial resistance to *β*-lactams and fluoroquinolones was observed. These resistance patterns among uropathogenic *E. coli* have important implications on the Baltic States. 

Resistance to penicillins was frequently observed in the study. Similar results have been demonstrated by studies conducted in Denmark and Estonia, where resistance to ampicillin was higher in strains isolated among uncomplicated and complicated UTI cases as well as pediatric UTI [31,32].

Notably, the highest resistance rate in *E. coli* was observed for cefuroxime (Table 1). High levels of resistance to cephalosporins in Lithuania impose a great threat to the population. Giedraitienė et al. demonstrated wide distribution plasmid encoded CTX-M class *β*-lactamases in Lithuanian hospitals [33]. Distribution of the CTX family of *β*-lactamases in clinical and veterinary sectors can possibly partly explain our observed resistance rates to cefuroxime, since the resistant strains can possibly circulate between both sectors. Considering the clinical importance of cephalosporins in Lithuania and high frequencies of resistance in both clinical and veterinary sectors, empirical therapy with oral cephalosporins, as a first-choice antibiotic, should be prescribed with a great caution.

The resistance to fluoroquinolones was also observed in this study. Alarmingly, 36% of isolates, obtained from pregnant women with ASB, showed high level resistance to ciprofloxacin indicating high distribution of resistance among strains of possibly commensal origin or colonization during frequent hospital visits. The levels of fluoroquinolone resistance among *Enterobacteriales* vary among European countries (8–10%) [34,35]. In our study, observed resistance to fluoroquinolones was higher in comparison to most of the European countries (average of resistance is 5.8%) [36]. However, similar levels of resistance to ciprofloxacin were observed by Stefaniuk et al. in Poland (34.2%) [37]. 

We demonstrated that among UTI-associated serogroups O15 was most prevalent and followed by *E. coli* O22 (Figure 2, Table 2). We have found that serogroups O15 and O22 were remarkably more resistant to ciprofloxacin (57.1% and 60%, respectively) and ampicillin (71.4% and 20.0%, respectively) (Table 2). Frequent occurrence and highly elevated resistance rates of O15 pose a potentially serious public health threat, as some globally distributed clonal variants of O15 are known to be extensively resistant and invasive [38,39,40]. We demonstrated that 42.8% of highly resistant O15 and 23.8% of O22 isolates were recovered from pregnant females with asymptomatic bacteriuria, suggesting a capability of resistant serovars to successfully colonize the host. Moreover, O15 and O22 were also prevalent among pyelonephritis cases; therefore, under certain conditions O15 and O22 can be responsible for complicated UTI and life-threatening conditions (Figure 2). 

Numerous studies have demonstrated that commensal, and often more resistant strains, fall to phylogenetic groups A and B1, whereas pathogenic, extraintestinal *E. coli* isolates belong to group B2 and, in some cases, D. We found that B2 and D group strains were also associated with ASB (28.0%), and followed by pyelonephritis (17.3%). However, these groups were more prevalent in unspecified UTI cases (22.7%) compared to A and B1 (12.0%). The study of Giedraitiene et al. analyzed the phylogenetic diversity of ExPEC in Lithuania. ExPEC isolates, obtained from the lower respiratory tract, the urinary tract, sterile body sites, wounds, and other body sites, demonstrated the predominance of B2 phylogenetic group (43.3%), followed by A, D, and B1 (28.9%, 27.8%, and 0%, respectively) [33]. Therefore, the diagnosis-specific phylogenetic composition can be seen in different types of infections caused by ExPEC.

Together this data shows the high prevalence of drug-resistant *E. coli* serogroups, pathogenic phylogenetic groups and profound antimicrobial resistance among Lithuanian patients with urinary tract conditions.

## 5. Conclusions

Due to the emergence and spread of drug-resistant *E. coli* serogroups in Lithuania and profound antimicrobial resistance to second-generation cephalosporins and fluoroquinolones, empirical therapy of such antimicrobials should be carefully reconsidered. Detailed molecular and epidemiological surveillance is needed to better understand virulence traits and resistance determinants among *E. coli* isolated from urinary tract conditions in Lithuania. 

## Figures and Tables

**Table 1:** Distribution of uropathogenic *Escherichia coli* (UPEC) resistance patterns among different study groups of the patients with urinary tract manifestations caused by UPEC.

Study Groups	No. of Isolates (%)	Distribution among Sexes		Frequency of Resistance (%)										
		No. among F (%) ^a^	No. among M (%) ^b^	AMP	AMC	CXM	CIP	AK	CN	TOB	F	TMP	IPM	MEM
Total isolates of *E. coli*	100 (100)	87 (87)	13 (13)	32 (32)	28 (28)	43 (43)	31 (31)	4 (4)	3 (3)	6 (6)	3 (3)	16 (16)	0 (0)	0 (0)
**Infectious group (total)**	53 (100)	40 (75.5)	13 (24.5)	19 (35.8)	15 (28.3)	26 (49.1)	15 (28.3)	2 (3.8)	1 (1.9)	4 (7.5)	1 (1.9)	6 (11.3)	0 (0)	0 (0)
Cystitis	10 (18.9)	9 (90)	1 (10)	3 (30)	1 (10)	5 (50)	2 (20)	1 (10)	0 (0)	1 (10)	0 (0)	1 (100)	0 (0)	0 (0)
Endometritis	2 (3.8)	2 (100)	0 (0)	2 (100)	0 (0)	0 (0)	0 (0)	0 (0)	0 (0)	0 (0)	0 (0)	1 (100)	0 (0)	0 (0)
Pyelonephritis	19 (35.8)	10 (52.6)	9 (47.4)	8 (42.1)	6 (31.6)	9 (47.4)	7 (36.8)	0 (0)	0 (0)	0 (0)	0 (0)	2 (10.5)	0 (0)	0 (0)
UTI with urosepsis	2 (3.8)	2 (100)	0 (0)	1 (50)	1 (50)	1 (50)	2 (100)	0 (0)	0 (0)	0 (0)	0 (0)	0 (0)	0 (0)	0 (0)
UTI ^c^	20 (37.7)	17 (85)	3 (15)	5 (25)	7 (35)	11 (55)	4 (20)	1 (5)	1 (5)	3 (15)	1 (5)	2 (5)	0 (0)	0 (0)
**Non-infectious group (total)**	32 (100)	32 (100)	0 (0)	8 (8)	8 (8)	11 (11)	12 (12)	1 (1)	2 (2)	2 (2)	0 (0)	6 (6)	0 (0)	0 (0)
ASB ^d^	32 (100)	32 (100)	0 (0)	8 (25)	8 (25)	11 (34.4)	12 (37.5)	1 (3.1)	2 (6.3)	2 (6.3)	0 (0)	6 (18.8)	0 (0)	0 (0)
**Unavailable clinical records (total)**	15 (100)	15 (100)	0 (0)	5 (5)	5 (5)	6 (6)	6 (6)	1 (1)	0 (0)	0 (0)	2 (2)	4 (4)	0 (0)	0 (0)
No clin. data ^e^	15 (100)	15 (100)	0 (0)	5 (33.3)	5 (33.3)	6 (40)	6 (40)	1 (6.7)	0 (0)	0 (0)	2 (13.3)	4 (26.7)	0 (0)	0 (0)

^a,b^ The data in this column indicates the distribution of uropathogenic *E. coli* isolates recovered from female (F) and male (M) patients; ^c^ symptomatic urinary tract infection (UTI) was considered when urine cultures were positive (<10^6^ CFU/mL) for *E. coli*; ^d^ asymptomatic bacteriuria (ASB) was considered when two consecutive urine cultures were positive (≥10^5^ CFU/mL) for *E. coli*; ^e^ isolates recovered with patients with known sex and with no detailed clinical data available. Abbreviations of antibiotics: AMP: ampicillin, AMC: amoxicillin/clavulanate, CXM: cefuroxime, CIP: ciprofloxacin, AK: amikacin, CN: gentamicin, TOB:tobramycin, F: nitrofurantoin, TMP: trimethoprim, IPM: imipenem, MEM: meropenem.

**Table 2:** Antimicrobial resistance patterns observed among different serogroups of uropathogenic *Escherichia coli* (UPEC) isolated from patients with urinary manifestations caused by UPEC.

*E. coli* Serogroups	No. of Isolates (%) per Group	Distribution of Serogroups among Sex		Frequency of Resistance (%)										
		No. among F (%) *^a^*	No. among M (%) *^b^*	AMP	AMC	CXM	CIP	AK	CN	TOB	F	TMP	IPM	MEM
Non-UTI associated *^c^*	36 (100)	29 (80.6)	7 (19.4)	0 (0)	16 (44.4)	35 (97.2)	1 (2.8)	2 (5.6)	0 (0)	5 (13.9)	0 (0)	3 (8.3)	0 (0)	0 (0)
UTI associated *^d^*	64 (100)	58 (90.3)	6 (9.4)	32 (50)	12 (18.8)	8 (12.5)	30 (46.9)	2 (3.1)	3 (4.7)	1 (1.6)	3 (4.7)	13 (20.3)	0 (0)	0 (0)
O2	1 (0.6)	1 (100)	0 (0)	0 (0)	0 (0)	1 (100)	0 (0)	0 (0)	1 (100)	0 (0)	0 (0)	0 (0)	0 (0)	0 (0)
O4	4 (6.2)	3 (75)	1 (25)	1 (25)	1 (25)	0 (0)	0 (0)	0 (0)	0 (0)	0 (0)	1 (25)	0 (0)	0 (0)	0 (0)
O8	4 (6.2)	4 (100)	0 (0)	2 (50)	1 (25)	0 (0)	1 (25)	0 (0)	0 (0)	0 (0)	0 (0)	2 (50)	0 (0)	0 (0)
O15	21 (32.8)	20 (95.2)	1 (4.8)	15 (71.4)	3 (14.3)	2 (9.5)	12 (57.1)	1 (4.8)	0 (0)	1 (4.8)	1 (4.8)	4 (19.0)	0 (0)	0 (0)
O16	3 (4.7)	3 (100)	0 (0)	2 (66.7)	0 (0)	1 (33.3)	2 (66.7)	0 (0)	1 (33.3)	0 (0)	0 (0)	0 (0)	0 (0)	0 (0)
O18	4 (6.2)	3 (75)	1 (25)	2 (50)	0 (0)	0 (0)	0 (0)	0 (0)	0 (0)	0 (0)	0 (0)	0 (0)	0 (0)	0 (0)
O22	15 (23.4)	13 (86.7)	2 (13.3)	3 (20)	2 (13.3)	1 (6.7)	9 (60)	0 (0)	1 (6.7)	0 (0)	0 (0)	0 (0)	0 (0)	0 (0)
O25	10 (15.6)	10 (100)	0 (0)	6 (60)	4 (40)	2 (20)	5 (50)	1 (10)	0 (0)	0 (0)	0 (0)	4 (40)	0 (0)	0 (0)
O83	2 (3.1)	1 (50)	1 (50)	1 (50)	1 (50)	1 (50)	1 (50)	0 (0)	0 (0)	0 (0)	1 (50)	1 (50)	0 (0)	0 (0)

*^a^* The data in this column indicates the distribution of uropathogenic *E. coli* isolates recovered from female (F) patients among different study groups; *^b^* the data in this column indicates the distribution of uropathogenic *E. coli* isolates recovered from male (M) patients among different study groups; *^c^* non-UTI associated serogroups are defined as all other possible *E. coli* O serogroups than the ones that can be detected using Li et al. [19] suggested multiplex PCR reaction; *^d^ E. coli* serogroups O1, O6, O7, O21, and O75 were not detected and therefore not included in the table. Abbreviations of antibiotics: AMP: ampicillin, AMC: amoxicillin/clavulanate, CXM: cefuroxime, CIP: ciprofloxacin, AK: amikacin, CN: gentamicin, TOB: tobramycin, F: nitrofurantoin, TMP: trimethoprim, IPM: imipenem, MEM: meropenem.

**Table 3:** Distribution of uropathogenic *Escherichia coli* (*E. coli*) phylogenetic groups A, B1, B2, and D among *E. coli* isolates obtained from female (F) and male (M) patients with urinary tract manifestations caused by *E. coli*.

Phylogenetic Groups	No. among F (%) *^a^*	No. among M (%) *^b^*	Total Number of Isolates per Group
Group A	7 (8.0)	0 (0)	7
Group B1	14 (16.1)	4 (30.8)	18
Group B2	43 (49.4)	7 (53.8)	50
Group D	23 (26.4)	2 (15.4)	25

*^a^* The data in this column indicates the distribution of uropathogenic *E. coli* isolates recovered from female (F) patients; *^b^* the data in this column indicates the distribution of uropathogenic *E. coli* isolates recovered from male (M) patients.

**Table 4:** Antimicrobial resistance patterns observed among different phylogenetic groups of uropathogenic *Escherichia coli* (UPEC) isolated from patients in Lithuania.

Phylogenetic Groups	Antimicrobial Resistance Profiles										
	AMP	AMC	CXM	CIP	AK	CN	TOB	F	TMP	IPM	MEM
Group A	3 (42.9)	1 (14.3)	4 (57.1)	2 (28.6)	0 (0)	0 (0)	1 (14.3)	0 (0)	2 (28.6)	0 (0)	0 (0)
Group B1	5 (27.8)	4 (22.2)	6 (33.3)	8 (44.4)	1 (5.6)	0 (0)	1 (5.6)	0 (0)	1 (5.6)	0 (0)	0 (0)
Group B2	15 (30)	11 (22)	20 (40)	14 (28)	1 (2)	3 (6)	2 (4)	2 (4)	6 (12)	0 (0)	0 (0)
Group D	9 (36)	12 (48)	13 (52)	7 (28)	2 (8)	0 (0)	2 (8)	1 (4)	7 (28)	0 (0)	0 (0)

AMP: ampicillin, AMC: amoxicillin/clavulanate, CXM: cefuroxime, CIP: ciprofloxacin, AK: amikacin, CN: gentamicin, TOB: tobramycin, F: nitrofurantoin, TMP: trimethoprim, IPM: imipenem, MEM: meropenem.

## Supplementary Materials

The following are available online at https://www.mdpi.com/1010-660X/55/11/733/s1, Table S1: The distribution of strains with intermediate resistance to tested antibiotic among Escherichia coli strains isolated from patients (*n* = 100) in Lithuania.
