# Supplementary material for: Molecular Characterization of Uropathogenic Escherichia coli Reveals Emergence of Drug Resistant O15, O22 and O25 Serogroups
Source: Medicina (Kaunas). 2019 Nov 11;55(11):733. doi: 10.3390/medicina55110733 (PMC6915421; doi:10.3390/medicina55110733)
Supplement: Supplementary file 1 [file medicina-55-00733-s001.pdf]

Table S1. The distribution of strains with intermediate resistance to tested antibiotic among *Escherichia coli* strains isolated from patients (n = 100) in Lithuania.

Antibacterial Agent	Abbreviation	Frequency of Intermediate Resistant Strains, *n* (%)
Ampicillin	AMP	2 (2)
Amoxicillin/Clavulanate	AMC	0 (0)
Cefuroxime	CXM	1 (1)
Ciprofloxacin	CIP	1 (1)
Amikacin	AK	6 (6)
Gentamicin	CN	2 (2)
Tobramycin	TOB	9 (9)
Nitrofurantoin	F	1 (1)
Trimethoprim	TMP	5 (5)
Imipenem	IMP	0 (0)
Meropenem	MEM	0 (0)
